# Dissipation of chlorantraniliprole, chlorpyrifos-methyl and indoxacarb—insecticides used to control codling moth (*Cydia Pomonella* L.) and leafrollers (*Tortricidae*) in apples for production of baby food

**DOI:** 10.1007/s11356-017-8821-z

**Published:** 2017-03-27

**Authors:** Ewa Szpyrka, Aneta Matyaszek, Magdalena Słowik-Borowiec

**Affiliations:** Laboratory of Pesticide Residue Analysis, Regional Experimental Station, Institute of Plant Protection - National Research Institute, Langiewicza 28, 35-101 Rzeszów, Poland

**Keywords:** Chlorantraniliprole, Chlorpyrifos-methyl, Indoxacarb, Dissipation, Apple, Baby food

## Abstract

Dissipations of three insecticides: chlorantraniliprole, chlorpyrifos-methyl and indoxacarb in apples were studied following their foliar application on apples intended for production of baby food. The apples were sprayed with formulations for control of codling moth (*Cydia Pomonella* L.) and leafrollers (*Tortricidae*). Six experiments were conducted; each insecticide was applied individually on dessert apples. A validated gas chromatography-based method with simultaneous electron capture and nitrogen–phosphorus detection (GC-ECD/NPD) was used for the residue analysis. The analytical performance of the method was satisfactory, with expanded uncertainties ≤36% (a coverage factor, *k* = 2, and a confidence level of 95%). The dissipations of insecticides were studied in pseudo-first-order kinetic models (for which the coefficient of determination, *R* ^*2*^, ranged between 0.9188 and 0.9897). Residues of studied insecticides were below their maximum residue limits of 0.5 mg/kg at an early stage of growth of the fruit. The half-lives of chlorantraniliprole, chlorpyrifos-methyl and indoxacarb were 16–17, 4–6 and 20–24 days, respectively. The initial residue levels declined gradually and reached the level of 0.01 mg/kg in 1 month for chlorpyrifos-methyl, 2 months for chlorantraniliprole and 2.5 months for indoxacarb. To obtain the insecticide residue levels below 0.01 mg/kg, which is the default MRL for food intended for infants and young children, the application of the studied insecticides should be carried out at recommended doses not later then: 1 month before harvest for chlorpyrifos-methyl, 2 months for chlorantraniliprole and 2.5 months for indoxacarb.

**Electronic supplementary material:**

The online version of this article (doi:10.1007/s11356-017-8821-z) contains supplementary material, which is available to authorized users.

## Introduction

The codling moth (*Cydia pomonella* L.) is considered one of the most important pests of apples in Poland and worldwide (Płuciennik 2013; Jones and Wiman 2008). In recent years, the climatic conditions have favoured a significant increase in a population of the codling moth (*Cydia pomonella* L.), as well as of other pests, such as leafrollers (*Torticidae*), in apple orchards. A rise in economic importance of this group of pests has been observed in Poland since 2000 (Płuciennik and Olszak 2006; Płuciennik and Olszak 2010).

In Poland, 24 plant protection products (ppp) containing 14 different active substances (a.s.) are recommended for protection of apples against the codling moth, and 13 ppp containing 10 different a.s. are recommended for the protection of apples against leafrollers. They include chlorantraniliprole, chlorpyrifos-methyl and indoxacarb, formulations belonging to three different chemical classes. These a.s. are widely used in European apple orchards and their residues are found in mature fruit (Scientific Report of EFSA 2015).

Chlorantraniliprole (IUPAC name 3-bromo-4′-chloro-1-(3-chloro-2-pyridyl)-2′-methyl-6′-(methylcarbamoyl)pyrazole-5-carboxanilide)) belongs to the anthranilic diamide group. This a.s. is used to control a broad spectrum of pests in a range of crops including potatoes, grapes, cabbage, apple and cotton (PPDB, Search engine). In Poland, chlorantraniliprole is a.s. present in only one ppp—Coragen 200 SC (200 g a.s. per 1 L of ppp)—used to control inter alia codling moth and leafrollers in apples.

Chlorpyrifos-methyl (IUPAC name *O*,*O*-dimethyl *O*-3,5,6-trichloro-2-pyridyl phosphorothioate) belongs to the organophosphate group. This a.s. is insecticide and acaricide used to control soil and foliage pests in grain, cotton, fruit, nuts and vegetables (PPDB). In Poland, this substance is a.s. in 2 ppp used on apples to control codling moth, apple sawfly, psyllids, aphids and leafrollers (Search engine).

Indoxacarb (IUPAC name methyl (*S*)-*N*-[7-chloro-2,3,4a,5-tetrahydro-4a-(methoxycarbonyl)indeno[1,2-e][1,3,4]oxadiazin-2-ylcarbonyl]-4′-(trifluoromethoxy)carbanilate) belongs to the oxadiazine group. This insecticide is used in a wide range of crops, including cotton, *Brassicas*; sweet corn, lettuce, fruiting vegetables and fruit including apples, pears and cherries, to control certain Lepidoptera, cockroaches and ants. In Poland, this substance is a.s. in 3 ppp used on apples to control the codling moth and leafrollers (Search engine).

The use of pesticides leads to the presence of their residues in fresh and processed food (Szpyrka et al. 2013; Szpyrka et al. 2015; Lozowicka et al. 2016). To ensure correct application of pesticides on agricultural crops and safeguard the health of consumers, maximum residue levels (MRLs) were established by authorities, for example, by the European Parliament and the Council of Europe (Commission Regulation (EC) No 396/ 2005). In the case of the most vulnerable group of people—infants and young children—special lower MRLs were established for pesticide residues. These MRLs, ranging between 0.004 and 0.008 mg/kg, apply to certain forbidden substances, whereas a threshold level of 0.01 mg/kg applies to the current-use pesticides (Regulation 2013a).

The main objective of this work was to study the residue concentration levels and dissipation kinetics over time for three insecticides used to control the codling moth (*Cydia Pomonella* L.) and leafrollers (*Tortricidae*): chlorantraniliprole, chlorpyrifos-methyl and indoxacarb, after their foliar application on apple trees, to determine the half-lives of these a.s. and intervals between their application and fruit harvest required to obtain the fruit suitable for production of baby food, i.e. with the residue levels below 0.01 mg/kg.

## Materials and methods

### Field experiment

In 2013–2016, six experiments were conducted in a commercial orchard of a land area of six hectares located in Rzeszów (south-eastern Poland, administrative division Województwo Podkarpackie). Standard agricultural practices, including pruning, fertilizing and soil management, were performed in the orchard during the growing seasons. Experiments were performed according to a randomized block scheme with four replications for each test, and each block consisted of four rows containing 200 plants in total. Apple trees were sprayed with Coragen 200 SC (a.s.—chlorantraniliprole) at a dose of 175 ml/ha, Reldan 225 EC (a.s.—chlorpyrifos-methyl) at a dose of 2.5 L/ha and Steward 30 WG (a.s.—indoxacarb) at a dose of 0.2 kg/ha (Table 1). Each insecticide was applied individually. Insecticides were applied with a sprayer Turbine N TNC 1000 (Italy). Sampling was performed sequentially during the pre-harvest periods. Each sample consisted of apples randomly chosen from a row of apple trees. The weight of collected samples of ripe apples was ≥1 kg, as required by the national regulation (Regulation 2013b).

**Table 1:** Plant protection products used in experiments

Plant protection product	Active substance / chemical group / concentration	Dose	Controlled pests	Mode of action on plant	PHI
Coragen 200 SC	Chlorantraniliprole / anthranilic diamide / 200 g/L (18.4%)	175 mL/ha	Codling moth, leafrollers	Surface and plunge	14
Reldan 225 EC	Chlorpyrifos-methyl / organophosphate / 225 g/L (19.29%)	2.5 L/ha	Codling moth, apple sawfly, psyllids, aphids, leafrollers	Surface and plunge	21
Steward 30 WG	Indoxacarb / oxadiazine / 300 g/kg (30%)	0.2 kg/ha	Codling moth, leafrollers	Surface	7

*PHI* pre-harvest interval

The weather conditions were monitored over the whole growing season by an automatic weather station (Institute of Meteorology and Water Management - National Research Institute, Jasionka, Poland). The average daily temperature and precipitations were recorded during the experiments (Fig. S1).

### Pesticide residue analysis

#### Reagents and standards

Acetone and diethyl ether were of p.a. grade, ACS+ISO+Ph. Eur (Honeywell Specialty Chemicals Seelze GmbH, Germany). Dichloromethane and petroleum ether were of per analysis grade, ACS+ISO (Honeywell Specialty Chemicals Seelze GmbH, Germany). Sodium sulphate (POCH, Gliwice, Poland) was baked at 550 °C for 7 h. Florisil (Sigma–Aldrich Sp. z o.o., Poznań, Poland) was activated by a bake-out at 130–135 °C for 7 h and then stored in a desiccator before use. A high-purity certified standards of insecticides were purchased from Ehrenstorfer (Augsburg, Germany). Stock solutions of approximately 1000 μg/mL in acetone were prepared. Intermediate standards (10 μg/mL) were prepared by dilution with acetone. The stock and the intermediate standards were stored at −16 °C. Working standards were prepared by diluting the intermediate standard with appropriate volumes of acetone (for spiking experiments) or with an extract of blank apple matrix (for GC calibration), and then stored at 4 °C.

#### Sample preparation

A Hallde VCB-62 blender (Hallde, Kista, Sweden) was used to homogenise the apple fruit samples (with stems removed). One hundred grams of each sample was weighed and stored at −16 °C before proceeding with sample preparation and gas chromatographic analyses according to the analytical method previously described in detail (Szpyrka and Walorczyk 2013). Briefly, an analytical sample (100 g) was homogenised with 150 mL of acetone for 2 min. The homogenate was filtered and an aliquot of filtrate, equivalent to 20 g of the analytical portion, was collected for further steps of the analysis. Following addition of 100 mL of 2.5% sodium sulphate in water, it was sequentially liquid–liquid partitioned with 20, 10 and 10 mL of dichloromethane. The combined extracts were rotary evaporated to dryness and dissolved in 10 mL of petroleum ether. An aliquot of 5 mL was transferred into a glass column packed with 1.1 g of activated florisil and 4.5 g of anhydrous sodium sulphate. The residues were eluted with 70 mL of diethyl ether/petroleum ether (3:7, *v*/*v*) and 70 mL of acetone/petroleum ether (3:7, *v*/*v*). The combined extracts were rotary evaporated and diluted with petroleum ether (10 mL). The final concentration of the sample extract was 1 g/mL.

The method for analysing pesticide residues was accredited by the Polish Centre for Accreditation (Polskie Centrum Akredytacji, PCA) according to the standard PN-EN ISO/IEC 17025:2005, with the certificate number AB 1279.

#### Gas chromatography analysis

A gas chromatograph, model 7890 (Agilent Technologies, Palo Alto, CA, USA), was used, equipped with a micro-electron capture detector (μECD) and a nitrogen–phosphorus detector (NPD). A HP-5 MS Ultra Inert capillary column, 30 m long × 0.32-mm I.D. × 0.25-μm film thickness (Agilent Technologies, Palo Alto, CA, USA), was used for the separations. The column was connected to μECD and NPD with a universal Y-splitter for simultaneous collection of chromatographic data from both detectors. Sample extracts (2 μL) were injected in splitless mode at 250 °C. The column oven temperature was programmed starting at 100 °C, then raised to 180 °C at 10 °C min^−1^ (maintained for 4 min), then raised to 220 °C at 3 °C min^−1^ (maintained for 15 min), and then raised to 260 °C at 10 °C min^−1^ (maintained for 11 min). The carrier gas was nitrogen (purity 6.0). Insecticides were determined with μECD at 280 °C with the makeup gas (nitrogen) flow rate of 30 mL/min. Confirmatory analyses were done using NPD at 300 °C with the hydrogen flow rate of 3 mL/min, air flow rate of 60 mL/min and makeup gas (nitrogen) flow rate of 10 mL/min. Agilent Technologies ChemStation Rev.B04.03 software was used for the instrument control, and data acquisition and evaluation.

#### Method validation

The method was validated before the determination of insecticide residues in real samples. Spiking recoveries of insecticides were determined at two concentration levels (with five replicates at each spiking level), using samples of untreated apples (Table 2). The method trueness and precision parameters, expressed as average recovery and relative standard deviation (RSD), were assessed against the acceptance criteria of EU SANTE/11945/2015 guidance document, specifying the required average recovery as ranging between 70 and 120% with associated RSD ≤ 20% for successful validation of the method for analysis of pesticide residues. The linearities of the calibration curves were verified over the concentration range, at five concentration levels, using matrix-matched calibration standards.

**Table 2:** Performance characteristics of the insecticide analysis in apples (*n* = 5)

Active substance	Spiking level (mg/kg)	Recovery ± RSD (%)	*U* (*k* = 2) (%)
Chlorantraniliprole	0.01 1.0	86.7 ± 7.3 92.8 ± 3.7	19.1 10.1
Chlorpyrifos-methyl	0.002 1.0	96.0 ± 3.1 92.9 ± 1.6	7.2 5.9
Indoxacarb 1	0.01 1.0	100.9 ± 12.2 70.8 ± 11.3	24.3 36.0
Indoxacarb 2	0.01 1.0	109.1 ± 8.9 83.1 ± 3.0	17.4 12.3

*RSD* relative standard deviation, *U* expanded uncertainty, *k* coverage factor, *n* number of replicates

To ensure reliability of the results for insecticide residues in apples, the method performance parameters were generated and assessed against the EU acceptance criteria before analyses of real samples were conducted. Table 2 shows the average recovery and RSDs data for insecticides obtained in spiking experiments. As it can be seen, the recoveries ranged from 70.8 ± 11.3% (indoxacarb 1 at a fortification level of 1.0 mg/kg) to 109.1 ± 8.9% (indoxacarb 2 at a fortification level of 0.01 mg/kg). RSDs for insecticide recoveries were ≤12.2%. These results were, therefore, considered satisfactory as they readily met the acceptance criteria in EU SANTE/11945/ 2015. The lowest spiking levels for which the validation criteria were satisfied were accepted as the limits of quantification (LOQs) of the method.

Very good linearities of the calibration curves over the concentration were obtained by using five concentration levels, yielding the coefficients of determination (*R* ^2^) exceeding 0.99. To overcome matrix effects and obtain more accurate quantification, all quantifications of insecticides in apple samples were performed using standards prepared in blank apple matrix extract.

Measurement uncertainty was estimated with the empirical model, using the data obtained in the validation experiments (recovery and RSD results). The expanded uncertainty of 5.9–36% (Table 2) was then calculated applying a coverage factor, *k* = 2, and a confidence level of 95%. These expanded uncertainty values were apparently below the maximum default value of ±50% recommended by the EU acceptance criteria in EU SANTE/11945/2015, thus proving the validated method was fit for purpose.

### Insecticide residue dissipation kinetics

The dissipation kinetics of insecticide residues were determined by plotting the residue concentration against the time elapsing from treatment; then, curves of best fit equations were determined, for maximum coefficients of determination (*R* ^2^). It was found that an exponential relationship existed for the insecticide dissipation in apples, corresponding to the general first-order kinetic Eq. 1:

1 $$ {R}_t={R}_0{e}^{- kt} $$

where *R*_*t*_ represents a concentration of pesticide residues at any time *t*, *R*_0_ is an initial residue concentration and *k* is the constant rate of the pesticide disappearance per day.

From this equation, the dissipation half-life period (*t*_1/2_ = *ln*(2)/*k*) of the pesticide and the time that must elapse until its residues reach the concentration level of 0.01 mg/kg (*t*_*R=0.01*_ = *ln*(0.01/*R*_0_)/(−*k*) were calculated.

## Results and discussion

**Fig. 1:**
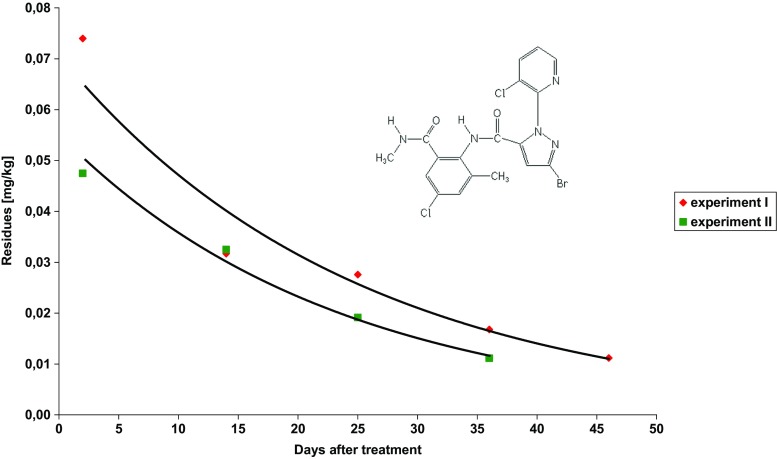
Dissipation trends for chlorantraniliprole in apple samples

**Fig. 2:**
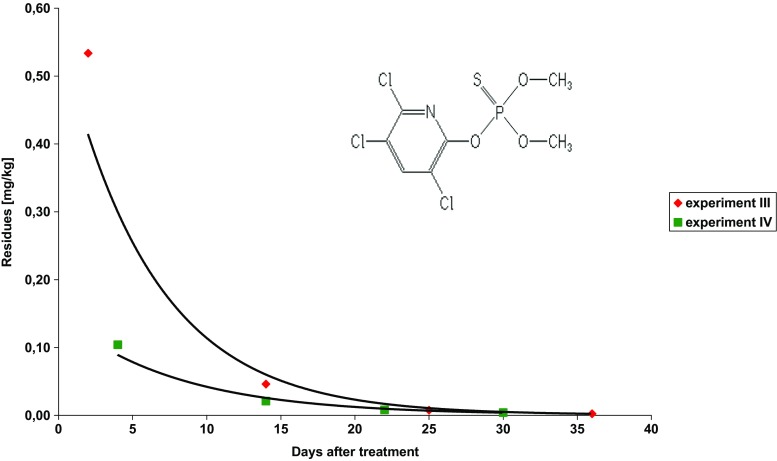
Dissipation trends for chlorpyrifos-methyl in apple samples

**Fig. 3:**
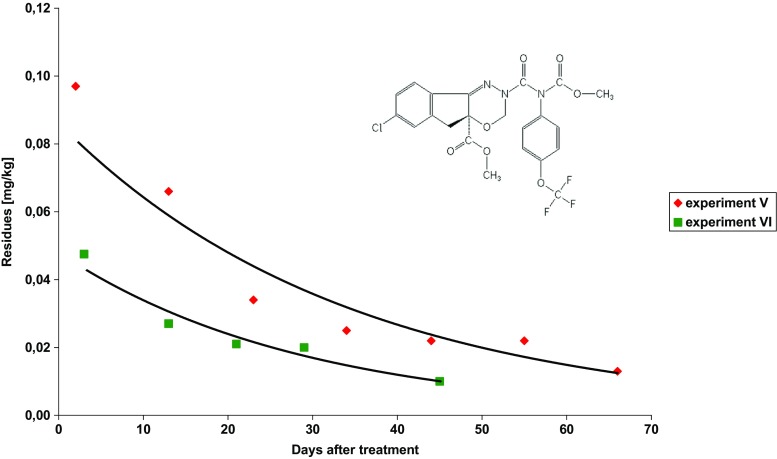
Dissipation trends for indoxacarb in apple samples

The study concerns dissipation of three insecticides used to control the codling moth (*Cydia Pomonella* L.) and leafrollers (*Tortricidae*): chlorantraniliprole, chlorpyrifos-methyl and indoxacarb, after their foliar application on apple trees. Each insecticide was applied individually. Examples of chromatograms obtained with both detectors are shown in Fig. S2. The insecticide dissipation patterns are presented in Figs. 1, 2 and 3. The mathematical models (exponential equations) matching the experimental data and describing the dissipation kinetics of insecticide residues yielded the regression fit, *R* ^2^, between 0.9188 and 0.9897. They are detailed in Table 3 along with half-lives (*t*_1/2_) and the times after which the residue concentration reached 0.01 mg/kg (*t*_*R*=0.01_).

**Table 3:** Parameters of insecticides dissipation in apples

Active substance	Experiment no.	Equation	*R* ^2^	*t*_1/2_ (day)	*t*_*R*=0.01_ (day)
Chlorantraniliprole	I II	*R*_*t*_ = 0.0703e^−0.0403*t*^ *R*_*t*_ = 0.0549e^−0.0431*t*^	0.9623 0.9897	17 16	48 40
Chlorpyrifos-methyl	III IV	*R*_*t*_ = 0.5728e^−0.1612*t*^ *R*_*t*_ = 0.1469e^−0.1258*t*^	0.9842 0.9819	4 6	25 21
Indoxacarb	V VI	*R*_*t*_ = 0.0858e^−0.0291*t*^ *R*_*t*_ = 0.0478e^−0.0345*t*^	0.9188 0.9598	24 20	74 45

*R* ^*2*^ coefficient of determination, *t*_*1/2*_ half-life, *t*_*R=0.01*_ time required for a residue concentration to reach 0.01 mg/kg

### Chlorantraniliprole—experiment I and II

Coragen 200 SC (chlorantraniliprole concentration—200 g/L) at dose of 175 mL/ha was applied on Jonagold Decosta (experiment I) and Gala (experiment II) varieties on June 10, 2015, in both tests. Laboratory samples were collected first on June 12, 2015, and then every 10–12 days. The average initial residue levels were 0.074 ± 0.013 mg/kg (experiment I) and 0.047 ± 0.017 mg/kg (experiment II). These residue levels were much below the MRL of 0.5 mg kg^−1^ (EU Pesticide database). The pre-harvest interval (PHI) of chlorantraniliprole is specified as 14 days. Insecticide dissipation patterns matched the exponential equations (Fig. 1). The initial levels of chlorantraniliprole residues dropped by half (*t*_1/2_) in 17 and in 16 days in the first and the second experiment, respectively. The concentration level of 0.01 mg/kg (the MRL applicable to baby food) was reached in 48 and in 40 days after the first and the second spraying with insecticides, respectively.

Chlorantraniliprole dissipations were studied in many crops but none of publications available concern apples. Half-lives of this a.s. amounted to 0.93–1.33 days in berseem (*Trifolium alexandrinum* L.) (Mandal et al. 2014), 1.31 days in cowpea fruit (Vijayasree et al. 2013), 1.36 in cauliflower (Kar et al. 2013), 1.58 and 1.80 in brinjal, 1.6 and 1.7 in okra fruit (Vijayasree et al. 2015), 2.441 days in summer season and 2.988 days in winter season in tomato (Shams El-Din 2015), 2.7 in grape (Malhat 2012) and 9.0–10.8 days in maize straw (He et al. 2016). In 2013, during the official control of pesticide residues in food, chlorantraniliprole was one of the most frequently detected pesticides in apples (10.3% samples), with the highest and the mean residue levels of 0.14 and 0.011 mg/kg, respectively (Scientific Report of EFSA 2015), that suggests its slower dissipation in apple fruit than in other crops, and is consistent with our results.

### Chlorpyrifos-methyl—experiment III and IV

Reldan 225 EC (chlorpyrifos-methyl concentration—225 g/L) at dose of 2.5 L/ha was applied on June 10, 2015, on Belle de Boskoop (experiment III) and on July 4, 2016, on Idared (experiment IV) varieties. Laboratory samples were first collected in 2 and 4 days after treatments, respectively, and then 8–12 days after treatments. The average initial residue levels were 0.534 ± 0.212 mg/kg (experiment III) and 0.104 ± 0.037 mg/kg (experiment IV). This difference in initial values of chlorpyrifos-methyl residues could result from differences in fruit size (an average weight of one apple in the first laboratory sample was 14.3 g in experiment III and 34.3 g in experiment IV). The PHI of chlorpyrifos-methyl is specified as 21 days and MRL is specified as 0.5 mg/kg (EU Pesticide database). Insecticide dissipation patterns matched the exponential equations (Fig. 2). The initial chlorpyrifos-methyl residue levels dropped by half (*t*_1/2_) in 4 and 6 days in the first and the second experiment, respectively. The concentration level of 0.01 mg/kg was reached in 25 and 21 days after the first and the second spraying with chlorpyrifos-methyl, respectively.

The half-lives of chlorpyrifos-methyl were 25 and 23 h on broad beans peel and pods, respectively (Ahmed et al. 2000), 1.31–1.60 days in brinjal fruit (Sawant and Dethe 2001), 0.64–4.7 days in apples from Hungary orchards (Ambrus and Lantos 2002) and 11.99 days in tomato fruit (Abbassy et al. 2015). Our results are correlated to those obtained by Ticha et al. (2007a, b), where in their two experiments chloropiryfos-methyl dropped to 0.01 mg/kg in about 28 days. In 2013, during the official control of pesticide residues in food, chlorpyrifos-methyl was detected in 0.7% of apple samples, with the highest and the mean residue levels of 0.39 and 0.012 mg/kg, respectively (Scientific Report of EFSA 2015).

### Indoxacarb—experiment V and VI

Steward 30 WG (indoxacarb concentration—300 g/kg) at a dose 0.2 kg/ha was applied on July 16, 2013, on Jonagold Decosta (experiment V) and on July 5, 2016, on Belle de Boskoop (experiment VI) varieties. Laboratory samples were first collected 2 and 3 days after treatments, respectively, and then every 8–16 days. The average initial residue levels were 0.097 ± 0.005 mg/kg (experiment V) and 0.048 ± 0.020 mg/kg (experiment VI). This difference in initial values of indoxacarb residue levels could result from precipitations on July 5 and 6, 2016. Initial residue levels were much below MRL of 0.5 mg/kg (EU Pesticide database). The PHI of indoxacarb is specified as 7 days. Insecticide dissipation patterns matched the exponential equations (Fig. 3). The initial indoxacarb residue levels dropped by half (*t*_1/2_) in 24 and in 20 days in the first and the second experiment, respectively. The concentration level of 0.01 mg/kg (the MRL applicable to baby food) was reached in 74 and in 45 days after the first and the second spraying with insecticides, respectively.

Indoxacarb dissipations were studies in cabbage, with a half-life of 1.92 and 2.88 days (Urvashi et al. 2012) and 2.8–4.6 days (Sun et al. 2012), in rice straw with a half-life of 5.83 days (Li et al. 2016) and in decaying cotton gin trash with a half-life of 26 days (Crossan and Kennedy 2008). There are no publications available concerning dissipation of indoxacarb in apple fruit. In 2013, during the official control of pesticide residues in food, indoxacarb was detected in 2.7% of apple samples, with the highest and the mean residue levels of 0.09 and 0.013 mg/kg, respectively (Scientific Report of EFSA 2015).

## Conclusions

The half-lives of insecticides tested varied from 4 to 6 days for chlorpyrifos-methyl to 16–17 days for chlorantraniliprole and 20–24 days for indoxacarb. Insecticides tested reached the level below MRL of 0.05 mg/kg much faster than their PHIs. For insecticide residues to reach levels below 0.01 mg/kg, being a default MRL for food intended for infants and young children, the studied insecticides should be applied at recommended doses 1, 2 and 2.5 months before harvest for chlorpyrifos-methyl, chlorantraniliprole and indoxacarb, respectively. This study is of high significance to apple producers in Poland, assisting them in an effective control of the most serious pests, the codling moth (*Cydia Pomonella* L.) and leafrollers (*Tortricidae*), while ensuring that problems related to the presence of pesticide residues are reduced to the minimum.

## Electronic supplementary material

Fig. S1 (DOC 35 kb)

Fig. S2 (BMP 3115 kb)

## References

[CR1] Abbassy MA, Nassar AMK, Salim YMM, Marzouk MA (2015). Toxic effects of residue amounts of chlorpyrifos-methyl in tomato to white albino rats. Research Journal of Environmental Toxicology.

[CR2] Ahmed MT, Loutfy N, Abdel Razik M, Hegazy ME, Hadidy FEL (2000). Residues of chlorpyrifos methyl and malathion on broad beans. Archiv fur Lebensmittelhygiene.

[CR3] Ambrus A, Lantos J (2002). Evaluation of the studies on decline of pesticide residues. J Agric Food Chem.

[CR4] Commission Regulation (EC) No 396/2005 of 23 February 2005 on maximum residue levels of pesticides in or on food and feed of plant and animal origin and amending Council Directive 91/414/EEC. *Off J Eur Union L 70/1*, 16.3.2005, with later amendments. Available at http://eur-lex.europa.eu/legal-content/EN/TXT/PDF/?uri=CELEX:32005R0396&from=en. Accessed April 2016

[CR5] Crossan AN, Kennedy IR (2008). Calculation of pesticide degradation in decaying cotton gin trash. Bull Environ Contam Toxicol.

[CR6] EU SANTE/11945/2015. (2015). Analytical quality control and method validation procedures for pesticide residues analysis in food and feed, pp 42. http://ec.europa.eu/food/plant/docs/plant_pesticides_mrl_guidelines_wrkdoc_11945_en.pdf. Accessed October 2016

[CR7] He M, Song D, Jia HC, Zheng Y (2016). Concentration and dissipation of chlorantraniliprole and thiamethoxam residues in maize straw, maize, and soil. J Environ Sci Health B.

[CR8] Jones VP, Wiman NG (2008). Longevity of the adult codling moth, Cydia pomonella, and the obliquebanded leafroller, Choristoneura rosaceana, in Washington apple orchards. J Insect Sci.

[CR9] Kar A, Mandal K, Singh B (2013). Environmental fate of chlorantraniliprole residues on cauliflower using QuEChERS technique. Environ Monit Assess.

[CR10] Li Z, Zhao X, Chen J, Wu Y, Zhang J, Zhang K, Hu D (2016). Determination of RH-5849 and indoxacarb in rice straw, rice husk, brown rice and soil using liquid chromatography-tandem triple quadrupole mass spectrometry following extraction with QuEChERS method. Biomed Chromatogr.

[CR11] Lozowicka B, Mojsak P, Jankowska M, Kaczynski P, Hrynko I, Rutkowska E (2016). Toxicological studies for adults and children of insecticide residues with common mode of action (MoA) in pome, stone, berries and other small fruit. Sci Total Environ.

[CR12] Malhat FM (2012). Determination of chlorantraniliprole residues in grape by high-performance liquid chromatography. Food Anal Methods.

[CR13] Mandal K, Kaur R, Sahoo SK, Arora R, Singh B (2014). Degradation pattern and risk assessment of chlorantraniliprole on berseem (*Trifolium alexandrinum* L.) using high performance liquid chromatography. Chemosphere.

[CR14] Płuciennik Z (2013). The control of codling moth (*Cydia pomonella* L.) population using mating disruption method. Journal of Horticultural Research 2013.

[CR15] Płuciennik Z, Olszak RW (2006). Wykorzystanie pułapek feromonowych do monitoringu owocówki jabłkóweczki i zwójkówek liściowych w sadach. Prog Plant Protect/Post Ochr Rośl.

[CR16] Płuciennik Z, Olszak RW (2010). Monitoring czterech gatunków zwójkówek liściowych w sadach z wy-korzystaniem pułapek feromonowych. Prog. Plant Protect./Post. Ochr. Rośl.

[CR17] PPDB: Pesticide Properties Data Base. University of Hertfordshire. Available at http://sitem.herts.ac.uk/aeru/ppdb/en/. Accessed October 2016.

[CR18] Regulation (2013a). Regulation (EU) No 609/2013 of the European Parliament and of the Council of 12 June 2013 on food intended for infants and young children, food for special medical purposes, and total diet replacement for weight control and repealing Council Directive 92/52/EEC, Commission Directives 96/8/EC, 1999/21/EC, 2006/125/EC and 2006/141/EC, Directive 2009/39/EC of the European Parliament and of the Council and Commission Regulations (EC) No 41/2009 and (EC) No 953/2009. *Off J Eur Union L 181/35*, 29.6.2013. Available at http://eur-lex.europa.eu/legal-content/EN/TXT/PDF/?uri=CELEX:32013R0609&from=EN. Accessed April 2016.

[CR19] Regulation (2013b). Regulation of the Minister of Agriculture and Rural Development from 27 November 2013 on the sampling of plants, plant products or other objects to test for the presence of residues of plant protection products. *Dz.U. z 2013 r. Nr 00, poz. 1549*. Available at http://isap.sejm.gov.pl/DetailsServlet?id=WDU20130001549. Accessed April 2016. [in Polish].

[CR20] Sawant NC, Dethe MD (2001). Dissipation of chlorpyrifos and chlorpyrifos-methyl on brinjal fruits. Pestology.

[CR21] Scientific Report of EFSA (2015). The 2013 European Union Report on pesticide residues in food. EFSA J.

[CR22] Search engine of plant protection products. Available at http://www.minrol.gov.pl/Informacje-branzowe/Wyszukiwarka-srodkow-ochrony-roslin. Accessed October 2016. [in Polish].

[CR23] Shams El-Din AM, Azab MM, Almaz MM, Gaaboub IA, Soliman HM (2015) Chlorantraniliprole behaviour in tomatoes under climatic changes of temperature and humidity. Egyptian Scientific Journal of Pesticides 1(2) Available at http://bu.edu.eg/portal/uploads/Agriculture/Plant%20Protection/1263/publications/Ali%20Mohamed%20Shams%20Eldin_Ibrahim%20Abdallha%20Gaaboub_www.esjpesticides.org.eg.pdf. Accessed December 2016

[CR24] Sun DL, Qiu J, Wu YJ, Liang HW, Liu CL, Li L (2012). Enantioselective degradation of indoxacarb in cabbage and soil under field conditions. Chirality.

[CR25] Szpyrka E, Walorczyk S (2013). Dissipation kinetics of fluquinconazole and pyrimethanil residues in apples intended for baby food production. Food Chem.

[CR26] Szpyrka E, Kurdziel A, Słowik-Borowiec M, Grzegorzak M, Matyaszek A (2013). Consumer exposure to pesticide residues in apples from the region of south-eastern Poland. Environ Monit Assess.

[CR27] Szpyrka E, Kurdziel A, Matyaszek A, Podbielska M, Rupar J, Słowik-Borowiec M (2015). Evaluation of pesticide residues in fruits and vegetables from the region of south-eastern Poland. Food Control.

[CR28] Tichá J, Hajšlová J, Kovalczuk T, Jech M, Honzicek J, Kocourek V (2007). Safe apples for baby-food production: survey of pesticide treatment regimes leaving minimum residues. Food Addit Contam.

[CR29] Tichá J, Hajšlová J, Kovalczuk T, Jech M, Honzicek J, Kocourek V (2007). Safe apples for baby-food production: survey of pesticide treatment regimes leaving minimum residues. Food Addit Contam A.

[CR30] Urvashi, Jyot G, Sahoo SK, Kaur S, Battu RS, Singh B (2012). Estimation of indoxacarb residues by QuEChERS technique and its degradation pattern in cabbage. Bull Environ Contam Toxicol.

[CR31] Vijayasree V, Bai H, Naseema Beevi S, Mathew TB, Kumar V, George T, Xavier G (2013). Persistence and effects of processing on reduction of chlorantraniliprole residues on cowpea fruits. Bull Environ Contam Toxicol.

[CR32] Vijayasree V, Bai H, Naseema Beevi S, Mathew TB, George T, Xavier G (2015). Persistence and effects of processing on reduction of chlorantraniliprole residues on brinjal and okra fruits. Environ Monit Assess.

