# Supplementary figures and images for: Dissipation of chlorantraniliprole, chlorpyrifos-methyl and indoxacarb—insecticides used to control codling moth (Cydia Pomonella L.) and leafrollers (Tortricidae) in apples for production of baby food
Source: Environ Sci Pollut Res Int. 2017 Mar 27;24(13):12128–35. doi: 10.1007/s11356-017-8821-z (PMC5410205; doi:10.1007/s11356-017-8821-z)

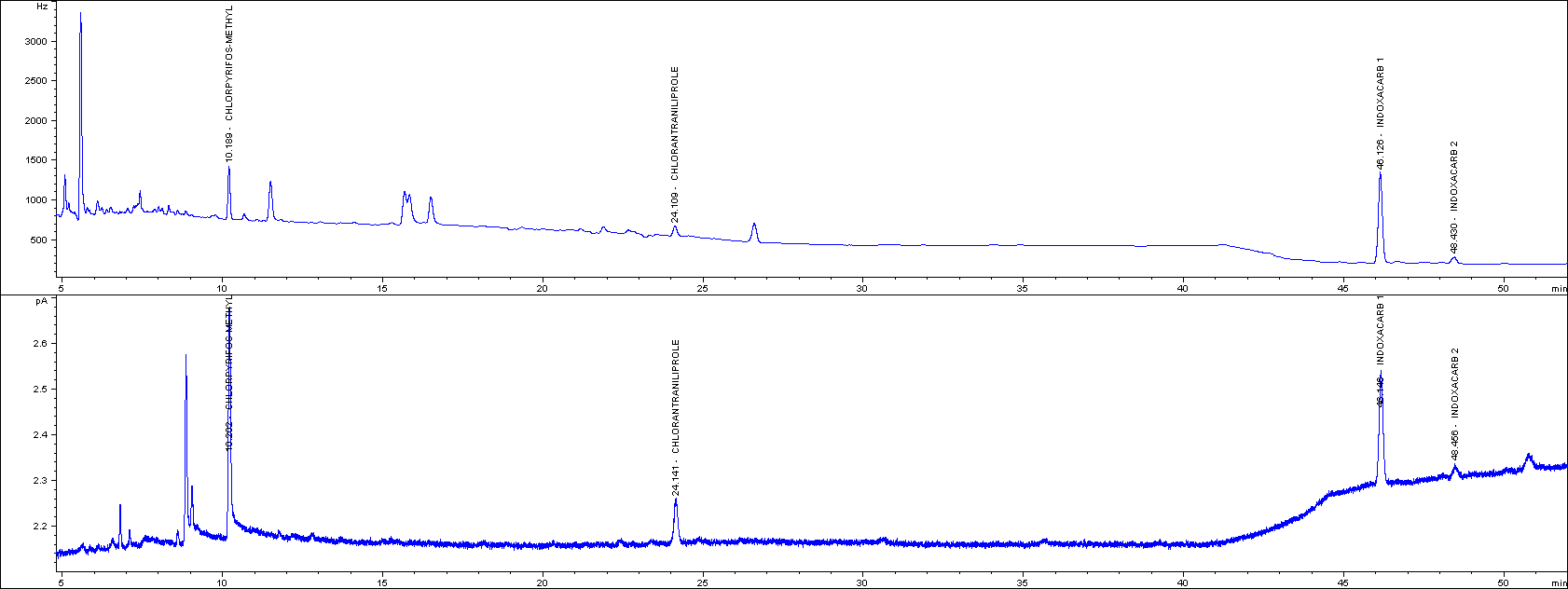

Supplement: Supplementary file 2 — (BMP 3115 kb) [file 11356_2017_8821_MOESM2_ESM.bmp]
