# Supplementary material for: Dissipation of chlorantraniliprole, chlorpyrifos-methyl and indoxacarb—insecticides used to control codling moth (Cydia Pomonella L.) and leafrollers (Tortricidae) in apples for production of baby food
Source: Environ Sci Pollut Res Int. 2017 Mar 27;24(13):12128–35. doi: 10.1007/s11356-017-8821-z (PMC5410205; doi:10.1007/s11356-017-8821-z)
Supplement: Supplementary file 2 [file 11356_2017_8821_MOESM1_ESM.doc]

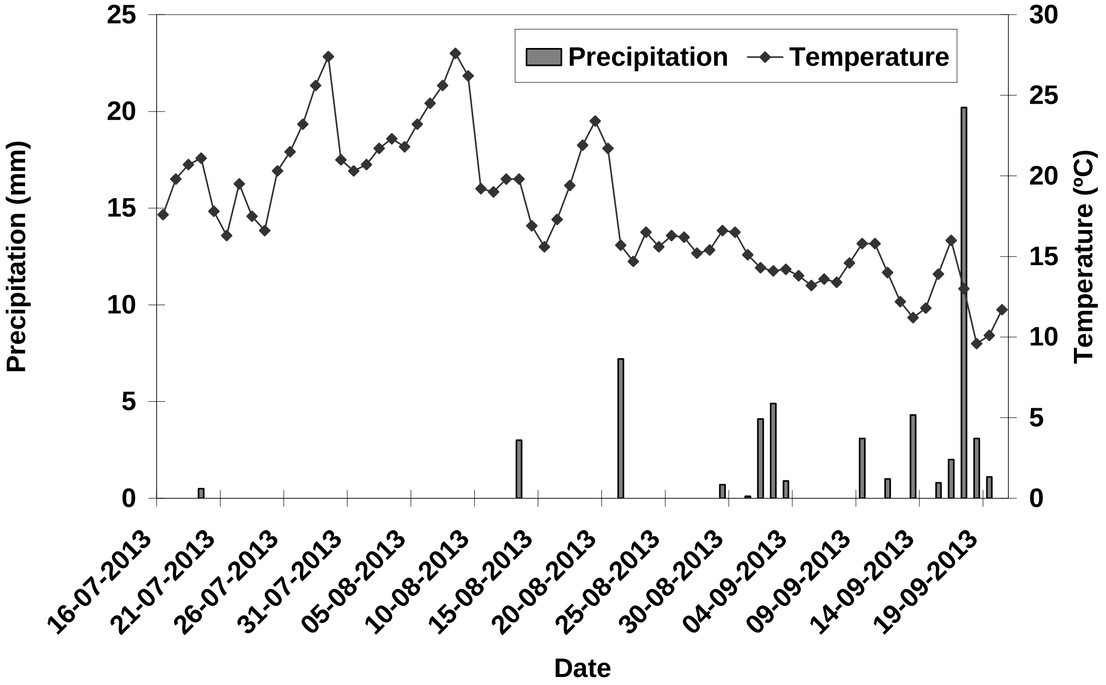
Precipitation
Temperature
Precipitation (mm)
Temperature (ºC)
Date
16-07-2013
21-07-2013
26-07-2013
31-07-2013
05-08-2013
10-08-2013
15-08-2013
20-08-2013
25-08-2013
30-08-2013
04-09-2013
09-09-2013
14-09-2013
19-09-2013

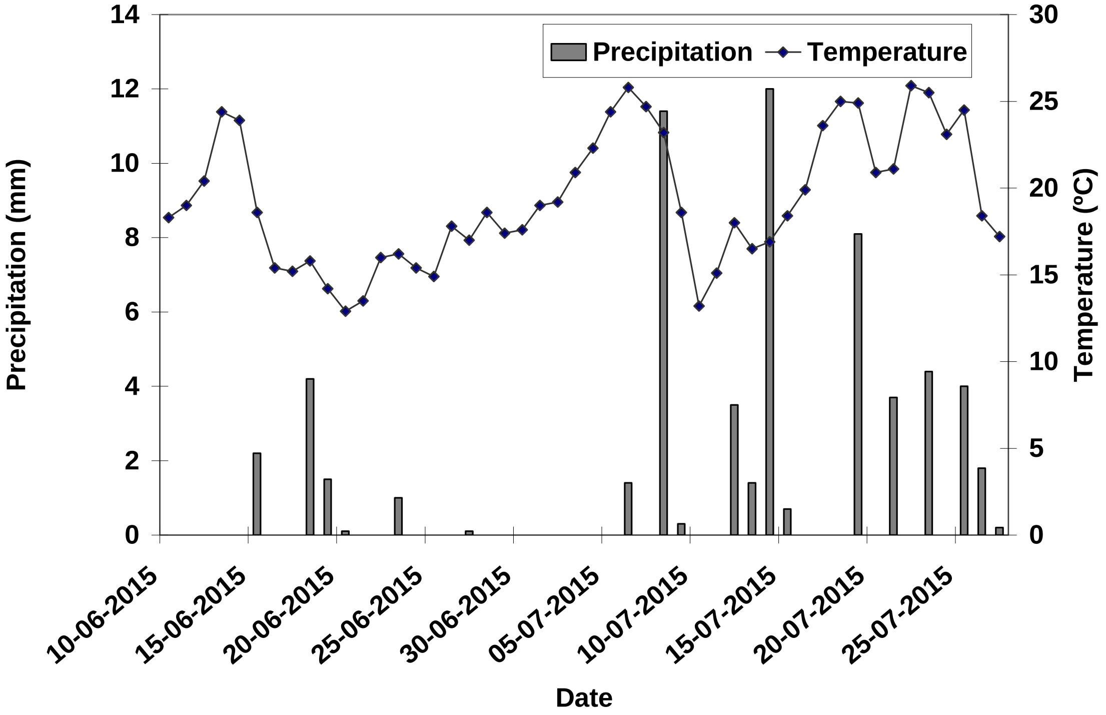
Precipitation
Temperature
Precipitation (mm)
Temperature (ºC)
Date
10-06-2015
15-06-2015
20-06-2015
25-06-2015
30-06-2015
05-07-2015
10-07-2015
15-07-2015
20-07-2015
25-07-2015

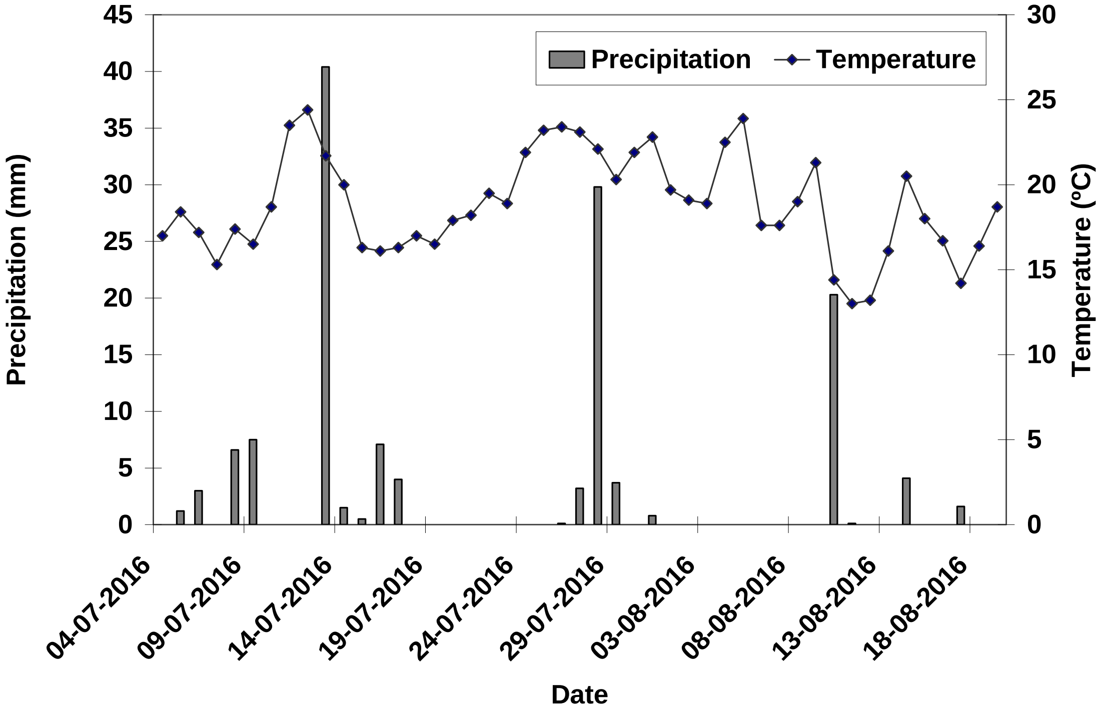
Precipitation
Temperature
Precipitation (mm)
Temperature (ºC)
45
40
35
30
25
20
15
10
5
0
30
25
20
15
10
5
0
04-07-2016
09-07-2016
14-07-2016
19-07-2016
24-07-2016
29-07-2016
03-08-2016
08-08-2016
13-08-2016
18-08-2016
Date
